# Giant seminoma presenting as a long standing hydrocele: a diagnostic challenge

**DOI:** 10.1093/jscr/rjag370

**Published:** 2026-05-24

**Authors:** Vishwas H M, Aishwarya Madasamy Swaminathan, Christopher Sam Thomas, Sunil Kumar Shetty, Jyothi K R

**Affiliations:** Department of General Surgery, Kasturba Medical College Mangalore, Manipal Academy of Higher Education, Manipal 576104, Karnataka, India; Department of Surgical Oncology, Manipal Hospitals, ITPL Main Road, Opposite Prestige Shantiniketan, KIADB Export Promotion Industrial Area, Whitefield, Bengaluru 560066, Karnataka, India; Department of General Surgery, Kasturba Medical College Mangalore, Manipal Academy of Higher Education, Manipal 576104, Karnataka, India; Department of General Surgery, Kasturba Medical College Mangalore, Manipal Academy of Higher Education, Manipal 576104, Karnataka, India; Senior Specialist, Department of General Surgery, Government Wenlock Hospital, Mangalore 575001, Karnataka, India

**Keywords:** seminoma, hydrocele, testicular tumor, orchidectomy, germ cell tumor

## Abstract

Seminoma usually presents as a painless solid testicular mass, but associated hydrocele may obscure the diagnosis and delay treatment. We report a 43-year-old man with progressive left scrotal swelling for 1 year and dull aching pain for 3 months. Clinical examination suggested hydrocele because the swelling was soft and transilluminant. Scrotal ultrasonography showed a bulky left testis with a solid component, raising suspicion for neoplasm. Contrast enhanced computed tomography demonstrated a heterogeneously enhancing enlarged left testis measuring 13 × 10.5 × 15 cm with moderate hydrocele. Serum beta human chorionic gonadotropin was elevated, whereas alpha fetoprotein and lactate dehydrogenase were within normal limits. The patient underwent high inguinal orchidectomy. Histopathology confirmed classical seminoma, staged as pT1b. This case highlights that long standing hydrocele with atypical features may conceal testicular malignancy, and early ultrasonography with tumor marker assessment is essential for timely diagnosis.

## Introduction

Testicular cancer accounts for ~1% of all male malignancies but is the most common solid malignancy in young and middle aged men [1]. Germ cell tumors comprise the vast majority of testicular neoplasms, and seminoma represents a major histological subtype [2]. Classical seminoma typically presents as a painless enlargement of the testis [3]. However, coexisting hydrocele can obscure the underlying testis on clinical examination and delay recognition of malignancy. We report a giant seminoma presenting as a long standing hydrocele, highlighting the importance of early imaging in apparently benign scrotal swellings with atypical features.

## Case report

A 43-year-old man presented with swelling of the left hemiscrotum for 1 year. The swelling was insidious in onset and gradually increased in size from ~3 × 3 cm to ~15 × 10 cm. He also reported a dragging, dull aching pain for 3 months. There was no history of trauma, fever, weight loss, urinary complaints, or previous scrotal surgery.

On examination, there was a large left scrotal swelling measuring ~15 × 10 cm. It was soft in consistency and transilluminant. The examiner was able to get above the swelling. The right testis was normal on palpation. No palpable inguinal or other regional lymphadenopathy was identified. The initial clinical impression was hydrocele.

Because the swelling was unusually large and the underlying testis could not be satisfactorily assessed, scrotal ultrasonography was performed. It demonstrated an anechoic fluid collection surrounding the left testis, along with a bulky testis containing a solid component, raising the possibility of an inflammatory or neoplastic lesion. Further evaluation with contrast enhanced computed tomography of the abdomen, pelvis, and scrotum showed a grossly enlarged heterogeneous left testis with heterogeneous post contrast enhancement, measuring ~13 × 10.5 × 15 cm, with associated moderate left hydrocele.

Laboratory investigations including complete blood count, liver function tests, and coagulation profile were within normal limits. Serum beta human chorionic gonadotropin was elevated, whereas alpha fetoprotein and lactate dehydrogenase were within normal limits.

**Figure 1:**
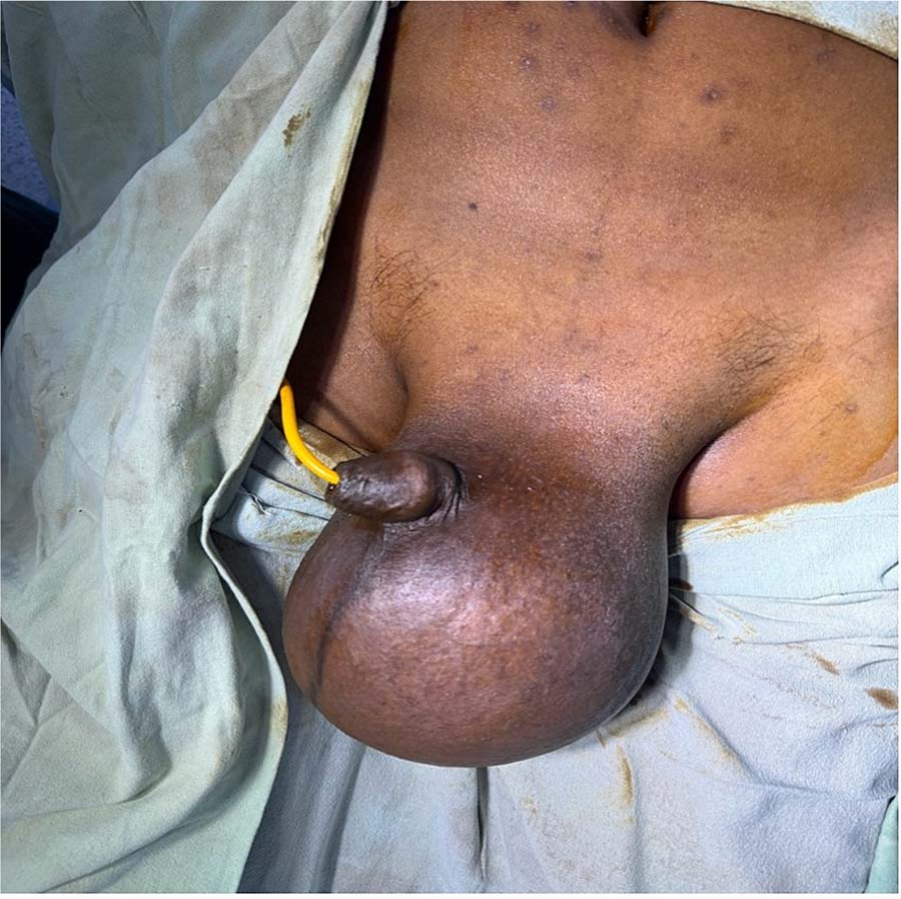
Preoperative clinical photograph showing marked swelling of the left hemiscrotum.

**Figure 2:**
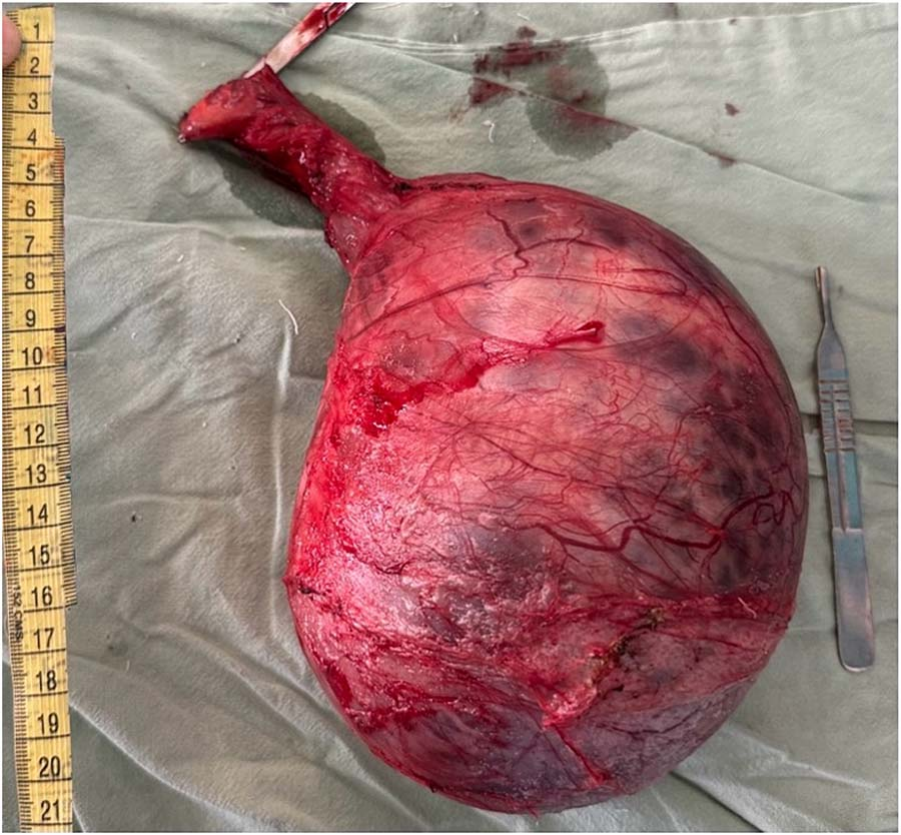
Gross specimen following left high inguinal orchidectomy showing an enlarged testicular mass.

**Figure 3:**
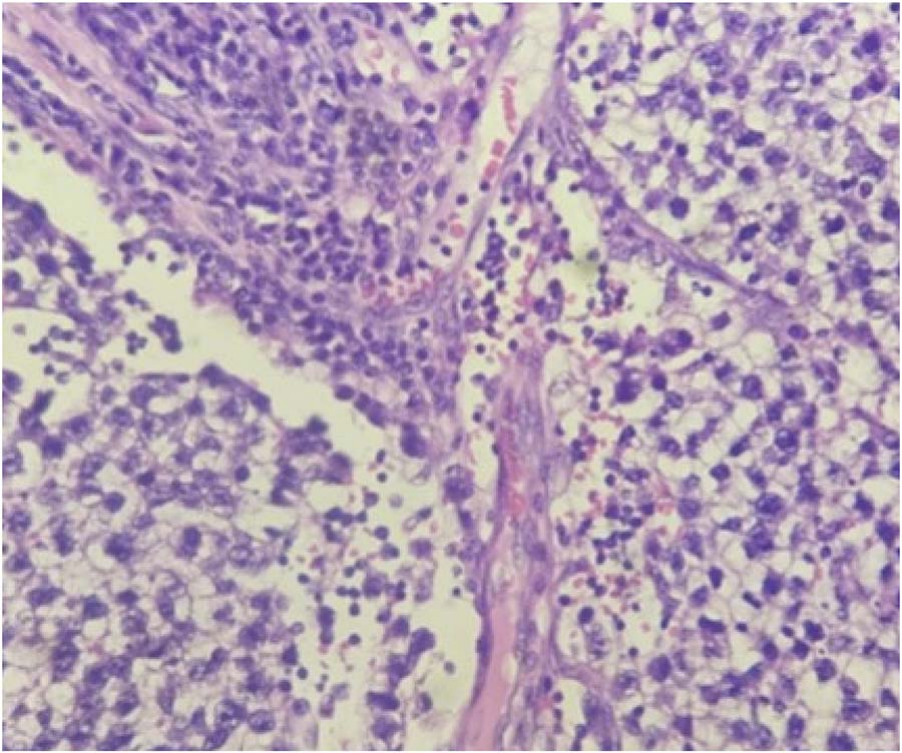
Histopathological image showing seminoma cells arranged in sheets and lobules separated by fibrous septae with lymphocytic infiltrates.

In view of the suspicion for testicular malignancy, the patient underwent left high inguinal orchidectomy (Fig. 1). Gross examination showed an enlarged testicular mass (Fig. 2). Histopathological examination demonstrated classical seminoma with tumor cells arranged in sheets and lobules separated by fibrous septae containing lymphocytic infiltrates (Fig. 3). The tumor was staged as pT1b. The postoperative course was uneventful. The patient was referred for oncological evaluation and adjuvant management planning, and remains on follow-up.

## Discussion

Seminoma is a well-recognized germ cell tumor with an excellent prognosis when diagnosed and treated early [2, 4]. The usual presentation is a painless unilateral testicular mass [3]. This case is clinically important because the tumor presented with features strongly suggestive of hydrocele, including a soft, transilluminant swelling, which could easily have led to further diagnostic delay.

Hydrocele is a common benign cause of scrotal swelling, but it may occasionally coexist with or mask a testicular tumor [3]. In such situations, reliance on clinical examination alone may be misleading, particularly when the testis is not distinctly palpable. This case reinforces the practical point that chronic hydrocele like swellings with progressive enlargement, pain, or inability to clearly assess the testis warrant prompt ultrasonography [5].

Scrotal ultrasonography remains the first line investigation for scrotal swellings because it reliably differentiates intratesticular from extratesticular pathology [5]. In our patient, ultrasonography was pivotal in identifying the solid intratesticular component that altered the diagnostic pathway from presumed benign hydrocele to suspected malignancy. Cross sectional imaging further delineated the size and morphology of the lesion [3].

The tumor marker pattern was also consistent with seminoma. Mild elevation of beta human chorionic gonadotropin may be seen in seminoma, whereas alpha fetoprotein is typically normal; elevated alpha fetoprotein should raise suspicion for a non-seminomatous component [2, 6]. Definitive treatment in suspected testicular cancer is radical inguinal orchidectomy, which allows both diagnosis and primary local treatment while avoiding scrotal violation [4, 7].

The added value of this report lies in its diagnostic lesson rather than its pathology alone. Although seminoma is common among germ cell tumors, a giant seminoma concealed by a long standing hydrocele is uncommon in routine practice and may mislead clinicians at first presentation. Early imaging of atypical hydrocele is therefore essential to avoid missing an underlying malignancy that remains highly curable when treated appropriately.

## Conclusion

Long standing hydrocele may conceal an underlying testicular malignancy and delay diagnosis. Progressive enlargement, pain, or inability to confidently palpate the testis should prompt immediate scrotal ultrasonography and tumor marker evaluation. This case highlights that seminoma can present deceptively as hydrocele, and that early recognition followed by high inguinal orchidectomy is crucial for optimal outcome.

## Data Availability

All relevant data supporting the findings of this case are included within the article. Further details are available from the corresponding author on reasonable request, subject to protection of patient confidentiality.
